# Validation of the animal model of bipolar disorder induced by Ouabain: face, construct and predictive perspectives

**DOI:** 10.1038/s41398-019-0494-6

**Published:** 2019-06-04

**Authors:** Samira S. Valvassori, Gustavo C. Dal-Pont, Wilson R. Resende, Roger B. Varela, Jéssica Lopes-Borges, José Henrique Cararo, João Quevedo

**Affiliations:** 1 Translational Psychiatry Laboratory, Graduate Program in Health Sciences, University of Southern Santa Catarina (UNESC), Criciúma, SC Brazil; 2 Translational Psychiatry Program, Department of Psychiatry and Behavioral Sciences, McGovern Medical School, The University of Texas Health Science Center at Houston (UTHealth), Houston, TX USA; 3 Center of Excellence on Mood Disorders, Department of Psychiatry and Behavioral Sciences, McGovern Medical School, The University of Texas Health Science Center at Houston (UTHealth), Houston, TX USA; 4 Neuroscience Graduate Program, The University of Texas MD Anderson Cancer Center UTHealth Graduate School of Biomedical Sciences, Houston, TX USA

**Keywords:** Neuroscience, Psychiatric disorders

## Abstract

A particular challenge in the development of a bipolar disorder (BD) model in animals is the complicated clinical course of the condition, characterized by manic, depressive and mixed mood episodes. Ouabain (OUA) is an inhibitor of Na^+^/K^+^-ATPase enzyme. Intracerebroventricular (ICV) injection of this drug in rats has been regarded a proper model to study BD by mimic specific manic symptoms, which are reversed by lithium (Li), an important mood stabilizer drug. However, further validation of this experimental approach is required to characterize it as an animal model of BD, including depressive-like behaviors. The present study aimed to assess manic- and depressive-like behaviors, potential alteration in the hypothalamic-pituitary-adrenal (HPA) system and oxidative stress parameters after a single OUA ICV administration in adult male Wistar rats. Moreover, we evaluated Li effects in this experimental setting. Data show that OUA ICV administration could constitute a suitable model for BD since the injection of the drug triggered manic- and depressive-like behaviors in the same animal. Additionally, the OUA model mimics significant physiological and neurochemical alterations detected in BD patients, including an increase in oxidative stress and change in HPA axis. Our findings suggest that decreased Na^+^/K^+^-ATPase activity detected in bipolar patients may be linked to increased secretion of glucocorticoid hormones and oxidative damage, leading to the marked behavioral swings. The Li administration mitigated these pathological changes in the rats. The proposed OUA model is regarded as suitable to simulate BD by complying with all validities required to a proper animal model of the psychiatric disorder.

## Introduction

Bipolar disorder (BD) is a psychiatric condition marked by aberrant mood swings, including mania, depression, and mixed states. Despite its significant impact in the life quality of the patients, both pathophysiology and pathogenesis of this disorder remain unclear^1–3^. In this regard, animal models are an essential strategy in search of data on behavioral status, neurotransmitter systems, and mechanisms underlying a particular mental disorder^4–6^. However, validation of an animal model for BD is difficult due to the complex clinical course of this condition^7^.

At present, most of the animal models for BD induces behaviors acutely mimicking a manic or depressive episode, not a combination thereof^4,7,8^. It is noteworthy that animal models of mental disorders should comply with the following requirements:^9^ Face validity, which indicates whether the model mimics the symptoms of a particular disorder; Construct validity, showing the ability of the model to simulate pathophysiological aspects of the illness; Predictive validity, which assesses whether the drugs included in therapy for the human condition can reverse the symptoms induced in the model.

The animal model of mania elicited by ouabain (OUA) – an inhibitor of Na^+^/K^+^-ATPase enzyme (EC 3.6.3.9), seems to meet these basic requirements, which renders it suitable to study certain behavioral and neurochemical aspects of BD^10–13^. OUA dose-dependently increases locomotor activity in rats, which is associated with manic-like behavior^12,14,15^. Intracerebroventricular (ICV) injection of this glycoside in rats also induces hypoactivity, which could be considered a depressive-like behavior. Such behavioral alterations are significantly prevented by administration of lithium (Li), an important mood stabilizer^13,16^. However, hypoactivity is not enough to mimic a state of depression, and additional experiments are necessary to validate this model. Besides, no research group showed manic- and depressive-like behaviors in the same animal after administration of OUA. The OUA model of mania was designed according to the “Na^+^/K^+^-ATPase hypothesis”, which hints decreased activity of the enzyme as a key factor to the outbreak of manic and depressive mood episodes in BD^14,17^. Indeed, several papers in the literature show that Na^+^/K^+^-ATPase activity is decreased in bipolar patients^17^. In addition, ICV administration of OUA in rats induces molecular changes similar to those detected in BD patients, neurotrophic factor alterations, mitochondrial dysfunction, and oxidative stress^12,18–20^.

Regarding the presented evidence, OUA ICV injection in rats could be a candidate to a putative experimental model of BD by reproducing specific pathophysiological mechanisms of the disorder and simulate the related mood swings^13,16^. However, further tests are required for the validation of the model. Therefore, the present study was designed to evaluate the behavior and potential Hypothalamic-Pituitary-Adrenal (HPA) axis alteration seven, nine and fourteen days after a single OUA ICV administration in Wistar rats; Li effects in this experimental context were also evaluated.

## Materials and methods

### Animals

Sixty day old male Wistar rats (*Rattus norvegicus*) were provided by the animal house at University of Southern Santa Catarina (UNESC). Animals were placed five per cage in a room with controlled conditions (temperature: 22 ± 1 °C; relative humidity: 45–55%), in a light/dark cycle of 12/12h (lights at 0600 hours). Rats also received chow and water. Experiments were performed according to the Guide for the Care and Use of Laboratory Animals^21^ and the guidelines of Brazilian Society for Neuroscience and Behavior, with approval by the UNESC’s Ethical Committee on Animal Use for Research (protocol # 66/2010).

### Surgery and OUA administration

Animals were submitted to anesthesia by intramuscular injection of ketamine and xylazine (80 mg/kg and 10 mg/kg body weight, respectively). Thereafter, skin covering rat skull was removed and a 27-gauge guide cannula (9 mm) was placed according to the following coordinates: posterior to bregma (0.9 mm); right from midline (1.5 mm); and above the lateral brain ventricle (1.0 mm). A 2 mm hole was made in the skull, through which a cannula was implanted ventrally to 2.6 mm of superior and fixed surface. On the fourth day after surgical procedure, animals received an ICV injection of artificial cerebrospinal fluid (aCSF) 5 μL alone or in combination with OUA (10^−3^ M)^14,17^. A cannula (30-gauge) was put inside guide cannula and linked to a microsyringe through a polyethylene tube. End of infusion cannula extended 1.0 mm beyond guide cannula aiming reach the right lateral brain ventricle.

### Experimental design

According to Diagnostic and Statistical Manual of Mental Disorders (5th edition), in bipolar patients, manic episodes last at least 7 days, whereas depressive symptoms are present within a 14 days period in the same subjects^22^. Thus, open field test (manic-like behavior assessment), sweet food consumption test and forced swimming test (depressive-like behavior assessment) were conducted in three stages: 7, 9, and 14 days after OUA injection, in order to evaluate potential manic-like behavior in early stage and depressive-like behaviors in a later stage of the model.

To test the predictive validity, on the day following the ICV administration, animals received saline (sal) or Li (47.5 mg/kg) by intraperitoneal injections for 14 days, twice a day^12^. Thus the animals were divided by simple randomization in four groups (I) aCSF + sal; (II) aCSF + Li; (III) OUA + sal; (IV) OUA + Li. (*n* = 8 rats per group per stage – 7, 9, and 14 days. Sample size was calculated by resource equation method, this sample size is enough to get significant results):

### Behavioral tests

The investigator were blinded to the group allocation during all the behavioral tests.

#### Open field test

In rodents, increased locomotor activity characterizes manic-like behavior^23^. Thus, locomotion was evaluated 7 or 14 days after ICV administrations through the open field test, according to the method described by Broadhurst^24^.

#### Forced swimming test

Procedure was carried out 14 days after injections, according to standardized method^25,26^.

#### Sweet food consumption

Anhedonia was assessed according sweet food consumption^27^. Experiment was conducted at 0800–1200 hours, 14 days following ICV administrations.

### Biochemical analysis

#### Samples

To test construct validity, at 7th, 9th, and 14th days after OUA injection, 24h after last administration of Li or sal, blood and brain samples were collected to biochemical parameter analysis (descried bellow). Immediately after behavioral tests, blood samples of the anesthetized with ketamine and xylazine rats were collected at 0800–1200 hours by cardiac puncture. To obtain serum, blood was centrifuged at 3000 × *g*, during 5 min, and then stored at −70 °C for subsequent analysis. Rats were submitted to euthanasia by decapitation immediately after collection of blood. The brain was excised and the cerebral structures (frontal cortex and hippocampus) were dissected, washed and stored at −70 °C.

#### Li levels in serum

Li levels were determined in serum samples of Li groups animals (aCSF + Li and OUA + Li) at the 7th, 9th, and 14th days according to Chapoteau and coworkers^28^.

#### Na^+^/K^+^-ATPase activity

The reaction mixture for Na^+^/K^+^-ATPase assay contained 5.0 mM MgCl2, 80.0 mM NaCl, 20.0 m MKCl, and 40.0 mM Tris–HCl, pH 7.4, in a final volume of 200 lL. The reaction was initiated by addition of ATP to a final concentration of 3.0 mM. Controls were carried out under the same conditions with the addition of 1.0 mM ouabain. Na^+^/K^+^-ATPase activity was calculated by the difference between the two assays according to the method of Wyse and coworkers^29^. Released inorganic phosphate (Pi) was measured by the method of Chan and coworkers^30^. Specific activity of the enzyme was expressed as nmol Pi released per min per mg of protein.

#### Analysis of HPA axis parameters

Adrenocorticotropic hormone (ACTH) and corticosterone levels were determined in serum samples of the three stages using radioimmunoassay-based kits developed by MP Biomedicals, LLC (Santa Ana, California, USA). Adrenal gland was excised and its weight then measured with a precision scale.

#### Evaluation of oxidative stress parameters in the cerebral structures

##### Lipid peroxidation

Activity of lipid hydroperoxide (LPH) was evaluated using a specific kit provided by Cayman (Cayman Chemical Company, Ann Arbor, Michigan, USA; catalog no. 705003). Thiobarbituric acid reactive species (TBARS) Assay Kit (Cayman; catalog no. 10009055) was used for the direct quantitative measurement of the malondialdehyde (MDA) level in the samples of brain tissue. 4-Hydroxy-2-nonenal (4-HNE) content was quantified using the kit provided by Cell Biolabs (Cell Biolabs, Inc., San Diego, California, USA; catalog no. STA-338). 8-Isoprostane content was measured using the ACE™ Competitive EIAs Kit (Cayman; catalog no. 516351) with 8-isoprostane-acetylcholinesterase (EC 3.1.1.7) as a tracer and 8-isoprostane specific antiserum.

Protein adducts arising from modification of cysteine, histidine or lysine residues by 4-HNE were quantified with the method of Kimura and coworkers^31^.

##### Protein oxidation parameters

Carbonyl group content was measured using a specific kit provided by Cell Biolabs (OxiSelect™ Protein Carbonyl ELISA Kit; catalog no. STA-310).

##### Activity of antioxidant enzymes

Another important parameter evaluated was glutathione peroxidase (GPx, EC 1.11.1.9) activity. GPx catalyzes the reduction of peroxides coupled to glutathione oxidation. Oxidized glutathione is newly reduced in a NADPH-dependent process, and NADPH consumption is followed by spectrophotometry at 340 nm wavelength as described by Wendel^32^. Finally, glutathione reductase (GR, EC 1.8.1.7) activity was determined on the basis of NADPH oxidation at 340 nm. Velocity of NADPH consumption reflects GR activity^33^.

### Protein levels

Protein content was measured to provide normalization for biochemical analysis, using as standard bovine serum albumin, according to method described by Lowry and coworkers^34^.

### Statistical analysis

Face validity results were evaluated by student’s *t* test and; Construct and predictive results were evaluated by Two-way analysis of variance (ANOVA) followed by Tukey’s for detection of differences between groups. Data were presented as mean ± standard deviation (SD). Software used in analysis was Statistica 7 (StatSoft, Inc., Tulsa, Oklahoma, USA). *p* ≤ 0.05 was required to rate differences as statistically significant.

## Results

### Face validity of the animal model of BD induced by OUA

**Fig. 1:**
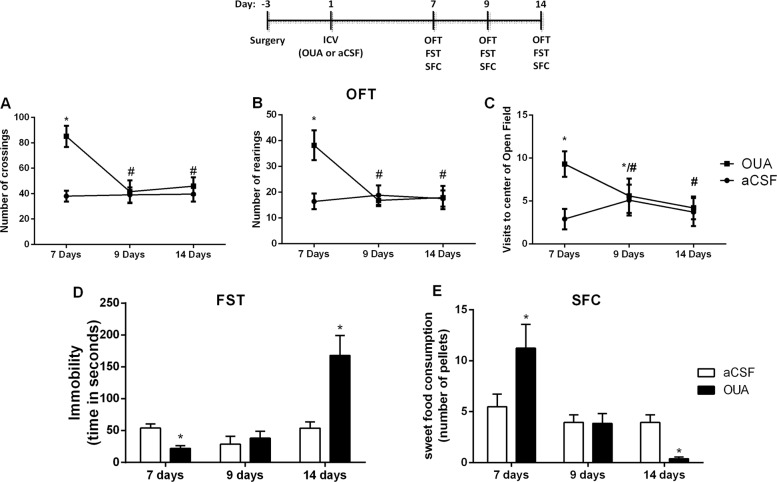
Effects of OUA on open field test (OFT), forced swimming test (FST) and sweet food consumption test (SFC) seven, nine and fourteen days after ICV injection in rats. Data are represented as means and standard deviation; *p < 0.05 compared to de aCSF group; #*p* < 0.05 compared to the OUA group, according to one-way ANOVA for repeated measures followed by Tukey’s post-hoc test (for OFT) or student’s *t* test (for FST and SFC)

The face validity of an animal model of psychiatric disorder is the ability of the model in mimic the symptoms of the disorder. It can be observed that seven days after a single OUA administration, the animals presented manic-like behaviors, which were demonstrated through increased crossings, rearings and visits to the open-field center. In the ninth day after OUA ICV administration, the same animals did not present locomotor (crossings), exploratory (rearings) or risk-taking behavior (visits to the open-field center) alterations (Fig. 1a–c). To evaluated depressive-like behavior, the animals were subjected to forced swimming and sweet food consumption tests (Fig. 1d, e). Nine days after OUA administration the animals did not present any behavioral alteration in the forced swimming or sweet food consumption tests when compared to the control group. Following the experimental design, fourteen days after OUA, the animals also did not show any alteration in the open-field test (Fig. 1a–c). However, fourteen days after OUA ICV injection, the animals increased the immobility time in the forced swimming test, which reflect a measure of behavioral despair. In the sweet food consumption test, a measurement of anhedonic-like behavior, rats OUA-administered reduced the sweet food pellet intake compared to the control group (Fig. 1d, e). It can be suggested that 14 days after OUA administration mimics depressive-like behaviors in rats. Together, these results demonstrate that a single injection of OUA induces manic- and depressive-like behaviors in the same animal, contemplating the face validity for a suitable animal model of BD.

### Predictive validity of the animal model of BD induced by OUA

**Fig. 2:**
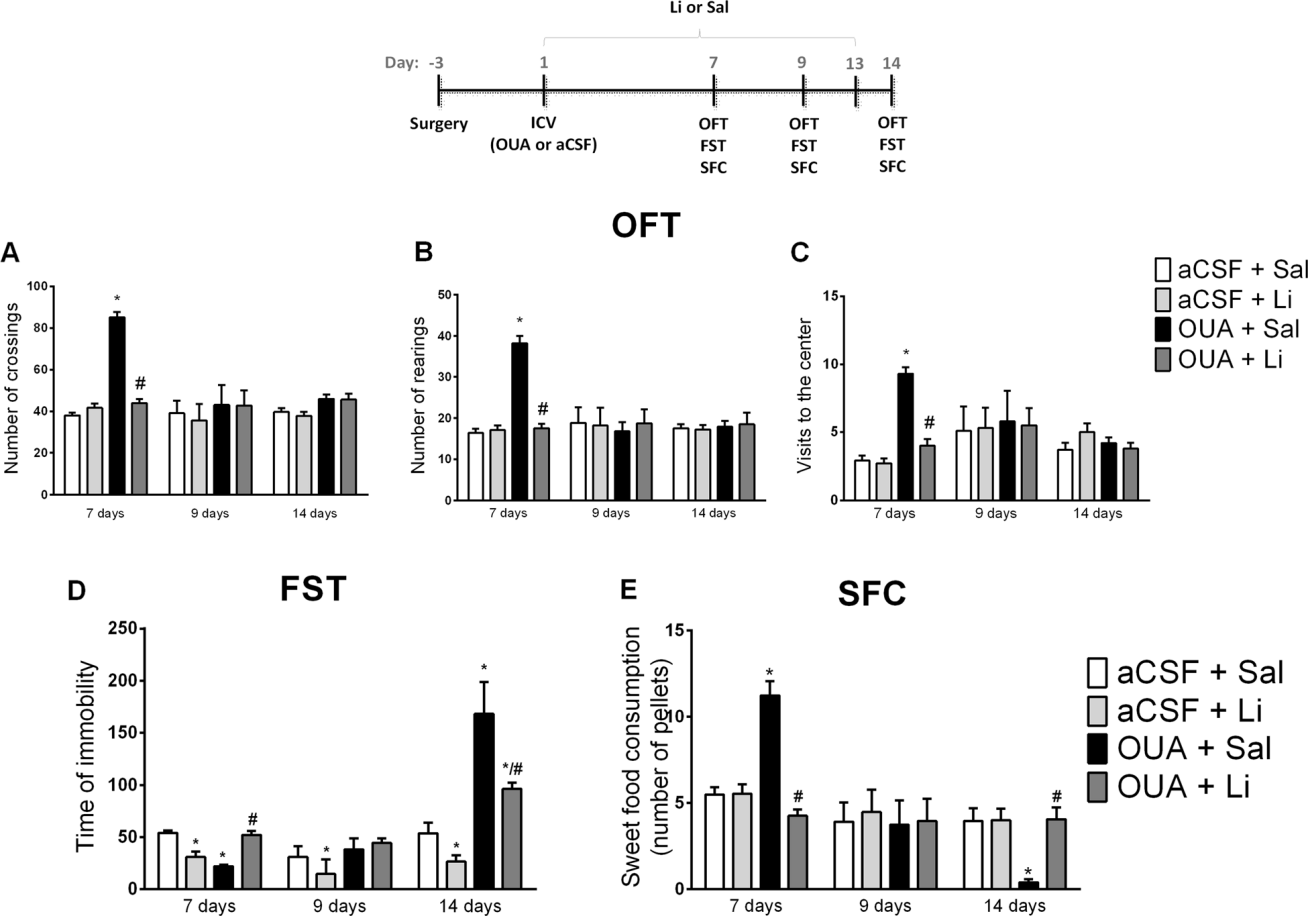
Effects of Li treatment on open field test (OFT), forced swimming test (FST) and sweet food consumption test (SFC) seven, nine and fourteen days after of OUA ICV injection in rats. Data are represented as means and standard deviation; *p < 0.05 compared to de aCSF + Sal group; #*p* < 0.05 compared to the OUA + Sal group, according to two-way ANOVA followed by Tukey’s post-hoc test

The predictive validity of the animal model of psychiatric disorder is the ability of the model in mimic the treatment of the disorder. As described previously in the literature^13^, Li reversed the increased in crossings, rearings, and visits to the open-field center induced by OUA seven days after ICV injection, mimicking the treatment of an acute manic episode (Fig. 2a–c). The rats treated with Li for nine days did not induce any behavioral alteration (open-field test, forced swimming and, sweet food consumption) after the ICV OUA injection, mimicking the maintenance of the treatment of BD (Fig. 2a–e). However, Li for nine days per se decreased the immobility time in the forced swimming test (Fig. 2d). Fourteen days of treatment with Li partially reversed the increased immobility time in the forced swimming test induced by OUA (Fig. 2d). Besides, Li reversed the decrease of food consumption induced by OUA (Fig. 2e), mimicking the treatment of a depressive episode. Therefore, it can be suggested that administration of Li in the OUA-administered rats mimics the treatment of bipolar disorder in both manic and depressive episodes, contemplating the predictive validity of the model. It is important to note that the plasmatic levels of Li of the animals treated with Li – 7, 9,and 14 days of treatment − were between 0.8 and 1.2 meq/L, which is the therapeutical dosage observed in bipolar patients (see Table 1).

**Table 1:** Lithium levels in serum of rats after 7, 9, and 14 days of OUA ICV injection

Levels of lithium (Li) in rat serum (mEq L serum^−1^)			
	7 days after	9 days after	14 days after
aCSF + Li	0.72	1.2	1.16
	0.85	0.84	0.85
	0.69	1.12	0.75
	0.75	0.96	0.95
	0.65	0.97	0.73
	0.71	0.85	0.83
	0.85	1.13	0.95
Mean ± S.D.	0.74 ± 0.077	1.01 ± 0.14	0.88 ± 0.15
Ouabain ± Li	0.92	1.12	0.74
	0.78	0.97	0.75
	0.69	0.92	1.12
	0.85	0.85	0.85
	0.96	0.96	0.75
	0.75	0.87	1.1
	0.81	0.85	0.95
Mean ± S.D.	0.82 ± 0.095	0.93 ± 0.096	0.89 ± 0.16

Data are presented in absolute values and total mean ± standard deviation

### Construct validity of the animal model of BD induced by OUA

**Fig. 3:**
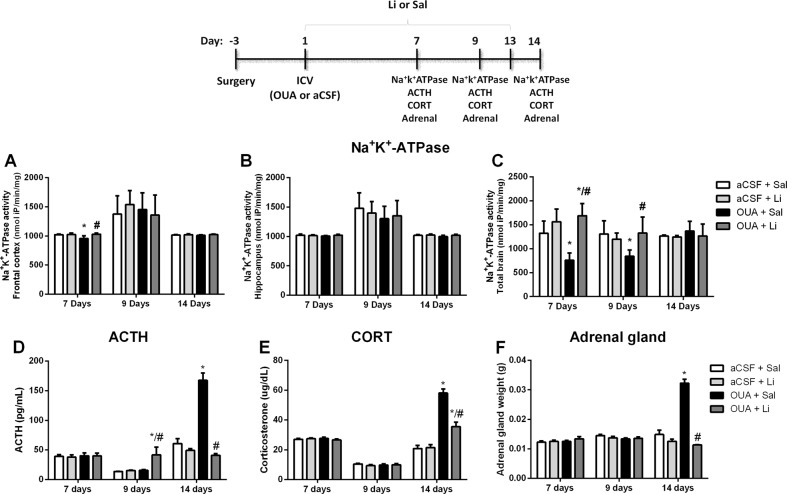
Effects of Li treatment on Na^+^/K^+^-ATPase activity in the brain, ACTH and corticosterone levels in serum and adrenal gland weight 7, 9, and 14 days after OUA ICV injection in rats. Data are represented as means and standard deviation; *p < 0.05 compared to de aCSF + Sal group; #*p* < 0.05 compared to the OUA + Sal group, according to two-way ANOVA followed by Tukey’s post-hoc test

The construct validity of the animal model of psychiatric disorder is the ability of the model in mimic some pathophysiologic alteration of the disorder. The development of the animal model of BD induced by OUA follow the hypothesis that decreased of Na^+^/K^+^-ATPase activity is the crucial factor in the trigger of manic and depressive mood episodes. Therefore, to confirm that the dose of OUA administered here decreases the Na^+^/K^+^-ATPase activity, this enzyme activity was evaluated in the brain of rats. The Na^+^/K^+^-ATPase activity decreased in the frontal cortex of rats seven days after OUA administration (Fig. 3a). In the total brain, Na^+^K^+^-ATPase activity decreased seven and nine days after OUA administration (Fig. 3c). The treatment with Li reversed the Na^+^K^+^-ATPase inhibition induced by OUA (Fig. 3a, c). No alterations were observed on Na^+^K^+^-ATPase activity in the hippocampus of the animals (Fig. 3b). Together these results demonstrate that ICV administration of OUA has construct validate because decreased the Na^+^/K^+^-ATPase activity in the brains, as observed in bipolar patients.

To increase the construct validity of the BD animal model induced by OUA, we evaluated biological markers alterations observed in bipolar patients, such as HPA axis and oxidative stress parameters. Fourteen days after OUA administration increased the levels of ACTH (Fig. 3d) and corticosterone (Fig. 3e) and increased the weight of adrenal gland of the rats (Fig. 3f), demonstrating alterations in the HPA. The treatment with Li reversed all alterations in the HPA axis induced by OUA (Fig. 3d–f). The ACTH levels increased in the group OUA + Li nine days after ICV administration (Fig. 3d). No HPA axis alteration was observed seven days after OUA administration.

**Fig. 4:**
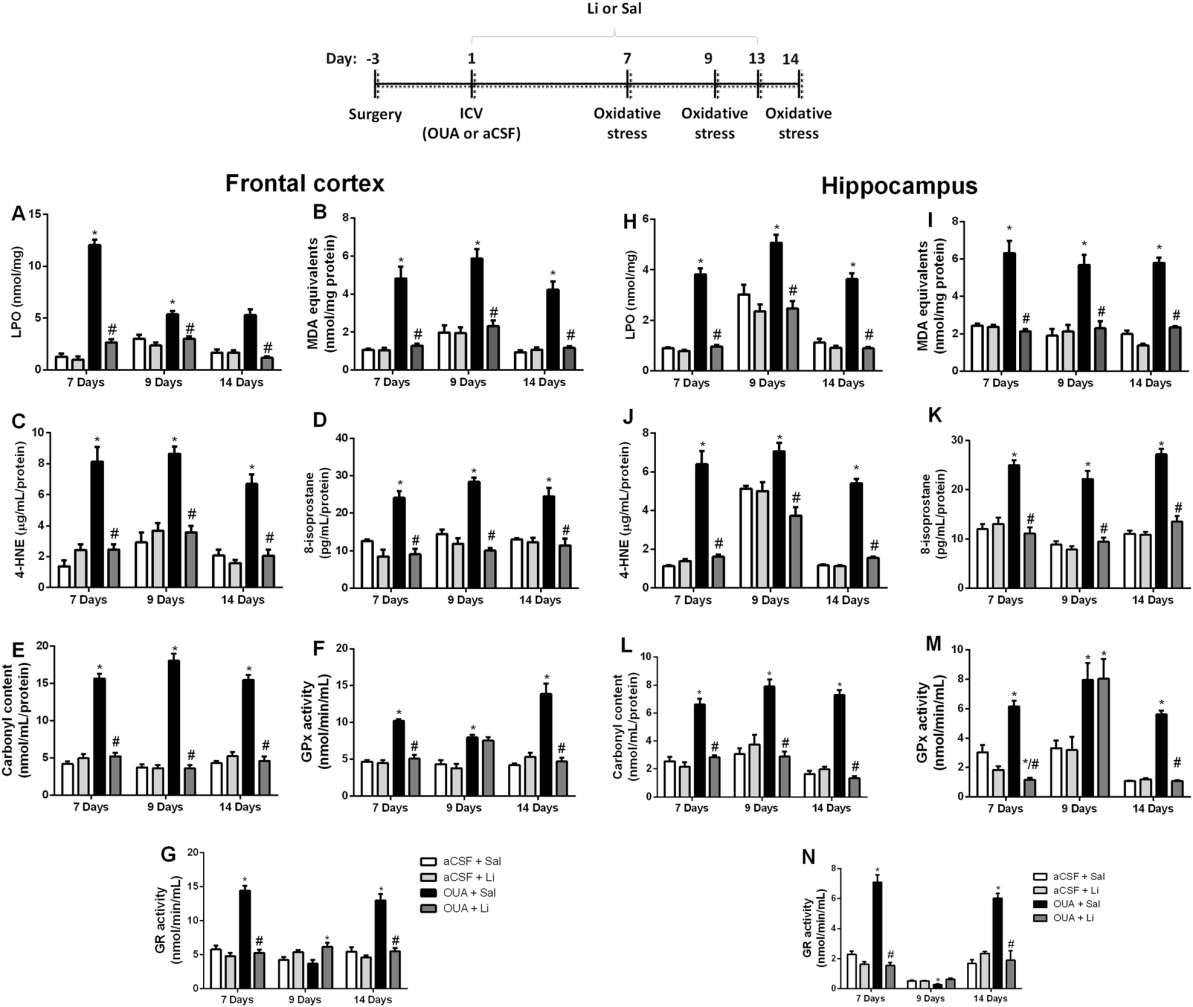
Effects of Li treatment on levels of LPO, MDA, 4-HNE, 8-ISO and carbonyl and GPx and GR activity seven, nine and fourteen days after OUA ICV injection in rats. Data are represented as means and standard deviation; *p < 0.05 compared to de aCSF + Sal group; #*p* < 0.05 compared to the OUA + Sal group, according to two-way ANOVA followed by Tukey’s post-hoc test

It is well described in the literature that bipolar patients have oxidative stress parameters altered in the brain; therefore, a suitable animal model of BD should present this biochemical alteration. In the present study, 7, 9, and 14 days after a single OUA administration induced an increase in the oxidative lipid damage in the frontal cortex and hippocampus of rats, which was evaluated through LPO (Fig. 4a, h), MDA (Fig. 4b, i), 4-HNE (Fig. 4c, j), and 8-ISO (Fig. 4d, k). An increase of protein carbonylation was observed in the frontal cortex and hippocampus of rats 7, 9, and 14 days after OUA administration (Fig. 4e, l). The treatment with Li reversed the oxidative damage to the lipid and protein induced by OUA, in both brain structures, and in all oxidative damage parameters evaluated (Fig. 4a–e, h–l). OUA ICV administration also alters antioxidant enzymes activities. Seven and fourteen days after ICV administration, OUA increased GR and GPx activities in frontal cortex and hippocampus, and Li reversed these enzymes alterations (Fig. 4f, g, m, n). However, 9 days after OUA administration increased the GPx activity in frontal cortex and hippocampus, but Li was not able to reverse this enzyme alteration. GR was not altered in any group nine days after ICV administration.

## Discussion

Recent genetic, neurobiological and pharmacological advances have contributed to the development of new animal models, which have been essential tools to studying intracellular systems that may be involved in the pathophysiology of psychiatric disorders^35,36^. However, no animal model completely mimics a particular psychiatric disorder. The development of an animal model of BD is a real challenge for researchers because of the complicated clinical course, which involves episodes of mania and depression^37^. It is important to emphasize that, at the moment, no pharmacological animal model mimic mania and depression in the same animal. In the present study, it was demonstrated that a single ICV injection of OUA induced manic- and depressive-like behaviors, contemplating the face validity for the consolidation of the animal model of BD.

The present study showed significantly increased locomotor activity 7 days after OUA injection in rats. Indeed, some papers also reported manic-like behaviors following OUA ICV administration in rats, including increased locomotor and exploratory activities^10,13,20,38^. In contrast, depressive-like behaviors (increased immobility time and decreased sweet food consumption) were observed in the animals 14 days after they received OUA. Li and colleagues^16^ demonstrated that OUA single injection significantly decreased motor activity in automated activity monitors, suggesting it as a model of bipolar depression. However, Li’s study did not demonstrate manic-like behavior at the same animal; besides, hypolocomotion could not be considered the best depressive-like behavior parameter. Differently, the present study showed anhedonic- and hopelessness-like behaviors (considered as standard depressive-like parameters in animals) after a single OUA injection. Thus, this study is the first one to demonstrate OUA-induced opposite behaviors in the same animal.

Nine days after OUA administration the animals did not present any behavioral alteration in the open field, forced swimming and sweet food consumption tests when compared to the control group. Therefore, it can be suggested that 9 days after OUA injection in rats mimics a euthymic episode. In contrast, Ruktanonchai et al.^39^. demonstrated OUA-induced hyperactivity in Sprague–Dawley rats persists for nine days. The discrepancy between the studies could be explained by the rat strains and experimental conditions differences. It is important to note that in the present study the protocol was repeated to biochemical analysis and we found the same in the open-field test, with no behavior alterations. The concept of euthymia does not include only the stable period with no mood alteration, but also an intra-state period when the patient do not present enough mood symptoms to be classified in a specific mood episode^40^. Thus, under our experimental conditions, it is possible to suggest that long-term OUA effects adequately fit the criteria of BD face validity, mimicking not only manic and depressive episodes but also an intra-state euthymia observed in BD patients.

The predictive validity of the animal model of psychiatric disorder is the ability of the model in mimic the treatment of the disorder. Li pretreatment for seven days prevented OUA-induced manic-like behaviors. There are several studies from our as well as other research groups demonstrating that Li treatment can reverse manic-like behavior in animals submitted to OUA ICV injection^10,13,20,38^. Herein, Li administration partly reversed the immobility time and completely reversed decreasing in the sweet food intake. Although some preclinical studies have previously described Li antidepressant effects^41,42^, this is the first study mimicking the maintenance treatment of depressive- and manic-like behaviors in a potential BD animal model. These data suggest that ICV OUA administration in the rat may prove the predictive validity for a BD animal model.

The construct validity of the animal model of psychiatric disorder is the ability of the model in mimicking some pathophysiologic alteration of the disorder. The development of the animal model of BD induced by OUA follow the hypothesis that decreased of Na^+^/K^+^-ATPase activity is the crucial factor in the trigger of manic and depressive mood episodes. Therefore, to confirm that the dose of OUA administered here decreases the Na^+^/K^+^-ATPase activity, this enzyme activity was evaluated in the brain of rats. It was observed that seven and nine days after ICV injection, OUA decreased Na^+^/K^+^-ATPase activity in the total brain of the animals. In the frontal cortex, the activity of this enzyme was decreased only seven days after OUA administration.

The hypothesis of Na^+^/K^+^-ATPase in the BD physiopathology was suggested more than 50 years ago^43,44^. Looney and El-mallakh^45^ demonstrated in a meta-analysis study that Na^+^/K^+^-ATPase activity is decreased in erythrocyte of BD patients. Since then, other studies keep contributing to this hypothesis^46–49^. Even a small reduction in this enzyme activity may lead the resting membrane potential close to threshold and then enhance neuronal excitability and impair Ca^2+^ depuration rate^50,51^. Increased neuronal excitability could trigger hyperactivity, which characterizes manic episodes in BD. However, long-term Na^+^/K^+^-ATPase inhibition, which increases neuronal excitability could lead to decreases the control of resting potential, difficulting the following neuronal depolarization. All these events may reduce neuronal signaling velocity and, consequently, decrease synaptic efficiency of neurons, leading to depressive episodes of BD^11,14^. Together these studies highlight the involvement of this enzyme on BD pathophysiology and reinforcing its relevance as a construct validity to animal models of this disorder.

To increase the construct validity of the BD animal model induced by OUA, we evaluated biological markers alterations observed in bipolar patients, such as HPA axis and oxidative stress parameters. The pattern of depressed behavior elicited by OUA in rats was concomitant to increases in adrenal gland weight, corticosterone, and ACTH serum level. Enlargement of the hypophysis and adrenal gland due to deregulated secretion of cortisol and ACTH (HPA axis activation) was detected in depressive patients^52–54^. Similarly, animals submitted to maternal deprivation/chronic stress exhibit increases in the weight of adrenal gland and serum content of ACTH and corticosterone, mimicking physiological alterations of depressive disorders in these models^55,56^. HPA axis activation in animals receiving OUA is ascribed to increased ACTH and corticosterone levels and adrenal gland hypertrophy. Also, Li also abrogated increases in the ACTH levels and adrenal gland weight induced by OUA. Once again, these findings support the OUA animal model to the intended aim, in compliance with the construct, face and predictive validities required to a suitable animal model for BD.

Manic-, euthymic- and depressive-like behaviors were accompanied by increased oxidative damage to macromolecules from rat frontal cortex and hippocampus, 7, 9, or 14 days after OUA ICV injection. Data show increased content of LPH, MDA (TBARS), 4-HNE and 8-ISO in these cerebral structures. Increased protein oxidation was also detected in similar conditions. Li administration completely counteracted such oxidative disturbances. Previous observations of our research group also showed increased MDA and carbonyl levels in rat frontal cortex and hippocampus, 7 days after OUA administration^13,19^. Such alterations were significantly prevented by Li in the paper carried out by Jornada and coworkers.13 In addition, increased carbonyl, 3-nitrotyrosine, 4-HNE, and 8-ISO contents were detected in the frontal cortex of BD patients in post mortem studies^57–59^. Significantly impaired Na + /K + -ATPase activity associated to lipid peroxidation in serum was also reported in BD patients, whereas these alterations were reversed by Li^60^. Thus, dysfunction of this enzyme activity probably is a pivotal mechanism in oxidative stress associated to the disorder. One of the therapeutic mechanisms of Li against oxidative damage could include enhancement of Na + /K + -ATPase activity. Data provided by the present paper show oxidative damage in rats receiving OUA, mimicking pathophysiological aspects in BD patients.

The present study also showed increased activity of GPx and GR in cerebral structures of rats receiving OUA, during the manic- and depressive-like behaviors; Li administration reversed these enzyme alterations. Partly in line with such observations, Andreazza and colleagues^61^ detected an increase in GR activity in blood of BD patients at late stages of the disorder. Besides, therapy with the glutathione precursor N-acetylcysteine in BD patients alleviated their manic and depressive symptoms^62^. Indeed, animal models of mania and depression have reported altered activity of antioxidant glutathione enzymes in the brain. To illustrate, a paper carried out using a depression model induced by immobilization stress showed increased activity of these enzymes in the hippocampus and cerebral cortex of rodents^63^, indicating that models of depression induce alterations in antioxidant enzymes resembling to that reported in the present study. However, Brocardo and coworkers^64^ showed decreased activity of GPx and GR in the same cerebral structures assessed here soon after OUA ICV injection in rats. Such discrepancy may be attributed to differences in the experimental designs between papers because we measured the activity of the antioxidant enzymes 7 or 14 days after OUA administration, aiming to simulate late stages of BD. Li administration prevented the deleterious modifications in GPx and GR elicited by OUA in cerebral structures of rats. Li was also implicated in the modulation of these enzyme activities in the brain of rats receiving amphetamine – a classic model of mania, potentially preventing oxidative damages in these animals^65^. Presented evidence hints alteration in the GPx and GR activities as a central factor for BD pathophysiology. One of the putative therapeutic roles of Li includes modulation of these antioxidant enzymes, collaborating to the maintenance of redox homeostasis in the brain.

Therefore, the OUA animal model comply with the three primary criteria (validities) required an adequate BD model. Since studies reported a close interaction between HPA axis and oxidative stress in BD patients^66,67^, reduced activity of Na^+^/K^+^-ATPase detected in these patients could be linked to increased secretion of glucocorticoid hormones and oxidative damage, leading to the mood swings. As a mood stabilizer drug, Li acts counteracting these pathological changes, which contributes to mitigating BD symptoms. Proposed OUA model could be used in studies on the pathophysiology of the disorder and to the screening of promising mood stabilizers drug candidates.
